# Characteristics of distance education interventions and related outcomes in primary school children during COVID-19 pandemic: A systematic review

**DOI:** 10.1371/journal.pone.0286674

**Published:** 2023-10-13

**Authors:** Hathairat Kosiyaporn, Mathudara Phaiyarom, Sonvanee Uansri, Watinee Kunpeuk, Sataporn Julchoo, Pigunkaew Sinam, Nareerut Pudpong, Rapeepong Suphanchaimat

**Affiliations:** 1 International Health Policy Program, Ministry of Public Health, Nonthaburi, Thailand; 2 Master of Public Health Program, Sirindhorn College of Public Health, Chonburi, Thailand; 3 Division of Epidemiology, Department of Disease Control, Ministry of Public Health, Nonthaburi, Thailand; Tallinn University: Tallinna Ulikool, ESTONIA

## Abstract

The COVID-19 pandemic containment measures such as school closures remarkably disrupt the educational system, from in-person learning to remote or distance education with different interventions. This study aimed to identify the characteristics of interventions in remote or distance education during the COVID-19 pandemic and evaluate the outcomes of each intervention. A systematic review was conducted between October 2021 and May 2022 using four databases. Finally, 22 studies met the eligibility criteria and were included for data analysis. Most of the interventions were synchronous student-centered approaches followed by asynchronous student-centered approaches and mixed-learning through online channels such as desktop- and web-based modality. Remote or distance education is effective in academic development in any learning approach while having mixed effects in student attitudes and perceptions. Academic-related behaviors were most engaged by students in synchronous student-centered approaches. Finally, difficulties or burdens, and mental health or social interaction were similar for all learning approaches in technological problems and support systems from families and teachers. Synchronous student-centered approaches should be the main method of education, but other approaches can be used to complement based on the students’ needs. Finally, educational infrastructure and support from teachers and parents are also necessary in remote or distance education. Further studies are needed to focus on primary school students, especially in low-income regions, and apply a randomized study design.

## Introduction

The novel coronavirus [COVID-19] was recognized as a global pandemic by the World Health Organization [WHO] and governments in each country including Thailand have implemented several measures to prevent transmission such as travel restrictions and closure of public spaces including school closure [1, 2]. Despite being an effective preventive measure, school closures did not only impact students’ health, but they also affected children’s learning for both short and long term [3]. School closure policies have been partially and fully implemented by many countries for more than 40 weeks since February 2020 [3]. It was found that about 90 percent of 188 countries had adopted online and/or broadcast remote learning policies [4] called ‘Emergency Remote Education’, which is an unplanned transition from traditional learning and teaching methods to remote ones in a state of emergency [5]. It can be adapted in online and offline platforms, with different pedological approaches and communication synchronicity [6].

Evidence has shown that COVID-19 and its policy responses have significant impacts on global education due to school closures and teaching transformations for long periods of time during the pandemic [4]. A systematic review of online learning during COVID-19 between 2019 and 2020 by Mohtar and Yunus [2022] addressed that only half of the findings [40 studies] revealed that students has engaged with online learning [7]. Accordingly, the academic performance of children has been negatively affected due to lack of contact hours and consultations with teachers when facing difficulties in learning/understanding [8]. Furthermore, children in poorer households that lack internet access, personal computers, television, or radio at home face learning inequalities [9]. For mental health, it was found that there was an increase of anxiety and loneliness in young children alongside child stress, sadness, frustration, indiscipline, and hyperactivity [10]. Overall, school closure policies with remote or distance education have different outcomes on learning engagement, academic achievement, access to educational resources, and mental well-being.

Although there are several advantages to remote or distance education, such as time and money savings and flexibility in learning methods, there are some challenges, such as a lack of communication and social interaction and complicated educational technology [11]. Accordingly, it emphasizes the urgency of remote or distance education’s impact evaluation during the COVID-19 pandemic, which tends to be unprepared. Moreover, remote or distance education is more likely to be an option integrating with in person learning even the COVID-19 pandemic disappears. Compared to other levels of education, the implementation of emergency remote or distance education is more challenging for primary school students, aged 6–12 years old. This is because they are still developing their self-regulation and attention control skills and are relatively technologically incompetent compared to students in secondary and tertiary education [11]. Previous systematic review studies by Bond [2021] and Crompton et al. [2021] about remote or distance education during COVID-19 specifically focused on secondary school students as the target population and mostly conducted in high-income countries [6, 12]. It also aimed to describe the technology used during the COVID-19 pandemic which was internet-based [6] and the tool typology were synchronous collaboration tools, knowledge organization and sharing tools, text-based tools, multimodal production tools, and social networking tools [6, 12]. Although the educational outcomes were systematically reviewed covering effects on academic performance, student engagement and educational inequality [13], the information of different distance educational interventions and its outcomes was sparse. Therefore, this study aims to identify the characteristics of interventions in remote or distance education during the COVID-19 pandemic and evaluate the outcomes of each intervention on socioemotional and behavioral changes, attitudes or perceptions, difficulties or burdens, and academic achievement among primary school children.

## Methods

This systematic review was conducted between October 2021 and May 2022 using the following protocol: setting operational definitions, a search strategy, and eligibility criteria, selecting studies, assessing quality and extracting data. The review followed the Preferred Reporting Items for Systematic Reviews and Meta-Analyses [PRISMA] [14], see S1 File.

### Operational definitions

The pedagogical approach includes teaching methods consisting of teacher- and student-centered approaches [15]. The teacher-centered approach involves the teacher playing the role of a master of a subject with little or no involvement from learners while the student-centered approach was the method that instructors play role as both teachers and learners [15]. Communication synchronicity is determined by the time of learning between teachers and learners; the synchronous approach occurs when teachers and learners are engaging at the same time whereas the asynchronous approach occurs at different times [16]. These definitions were applied to thematize data in different types of educational intervention linking with its outcomes in the data analysis part.

### Search strategy

The search terms were developed in three domains: a] COVID-19; b] primary education; and c] remote or distance education with exclusion of higher education, adult learning, health professional education, special education for children with disability, and physical health [see the details of each search term in Table 1]. These were applied in four databases: PubMed, Scopus, Web of Science, and EBSCOHOST due to the database’s coverage in public health and education. The limitation of English literature, journal article and timeline from 2020 to 2021 will be used.

**Table 1:** Search terms.

Domains	Search terms
COVID-19	covid* OR corona* OR “SAR-Cov*” OR CoV* OR “2019-nCoV” OR “n-CoV”
	AND
Primary education	“elementary school” OR “middle primary” OR “upper primary” OR “primary school” OR student* OR pupil* OR child* OR kid*
	AND
Remote or distance education	“distance education” OR “distance learning” OR “online education” OR “online learning” OR “remote learning” OR “remote education” OR “remote schooling” OR “virtual learning” OR “virtual education”
	NOT
	universit* OR “higher education” OR postgrad* OR undergrad* OR “tertiary education” OR college OR campus* OR dent* OR nurs* OR pharmac* OR medic* OR "health professional*" OR "health worker*" OR "healthcare worker*" OR "health personnel" OR clinic* OR patient* OR disabilit* OR physical activit* OR nutrition OR diet*

### Eligibility criteria

Inclusion and exclusion criteria were applied to ensure article relevance to the study objectives by using the PICO strategy as shown in Table 2. The inclusion criteria comprised of studies that involved children aged 6 to 12 years or are in primary schools, remote or distance education during a public health emergency, and intervention outcomes such as socioemotional and behavioral changes. The documents included in this study were peer-reviewed literature from primary research, published in English language between 2020 and 2021 [COVID-19 pandemic period], and with retrievable full-text articles. The studies related to higher education, adult learning, health professional education, special education for children with disability, and physical health outcomes were excluded as same as grey literature and articles in other languages. Empirical studies were included covering quantitative and qualitative studies which would be reflecting the objective and subjective educational outcomes.

**Table 2:** Inclusion and exclusion criteria.

Inclusion criteria		Exclusion criteria
**Population**	Children aged 6 to 12 years or in primary school	Those who are in higher education, adult learning, health professional education, special education for children with disability
**Intervention**	Remote or distance education during a public health emergency including online- and offline-based methods	-
**Comparison**	None	None
**Outcome**	Primary outcomes • Socioemotional and behavioral changes • mental health/social interaction • academic-related behaviors e.g. engagement • attitudes/perceptions • difficulties/burdens Secondary outcomes • academic achievement	• Physical health • Health issues not related to education [e.g., handwashing]
**Study design**	Empirical study • Randomized controlled trial study • Quasi-experimental study • Cohort study • Cross-sectional study • Case-control study • Qualitative study • Mixed-method study	Non empirical study • Commentary/Perspective/Opinion • Letter • Editorial • Review • Protocol
**Others**	• Studies published in English • Peer-reviewed studies • Studies related to COVID-19 pandemic [2020–2021] • Studies with full-text retrieval	-

### Study selection

Six researchers [HK, MP, SU, WK, SJ, PS] were responsible for title, abstract and full-text screening, and quality assessment. Groups of two or three researchers independently screened titles and abstracts first; if there was a disagreement among them, they would discuss to reach a consensus. The same process was conducted for the quality assessment and full paper review.

### Data analysis

Data analysis had two parts: quality assessment and data extraction. Independent data analysis was individually undertaken by researchers. If there was a disagreement or unclear information, a consensus would be reached among researchers to ensure data accuracy.

#### Quality assessment

The Joanna Briggs Institute [JBI] critical appraisal tools were used to assess quality of each full-text article [17]. This appraisal covered the ethical consideration and possibility of bias in data collection such as inclusion and exclusion criteria and loss to follow up, data measurement and data analysis [17]. According to different study designs, such as quasi-experimental study and cross-sectional study, the quality of each study type was separately assessed following the JBI critical appraisal tools. Although the JBI tool was developed to assess study in qualitative aspects, the quality score in percentage was also applied to evaluate overall quality and the cut-off point of acceptable quality was set at more than 50% [18].

#### Data extraction

Data related with these three themes was analyzed including: **a] characteristics of studies**—author, title, year of publication, objective of study, country, study design, target groups, settings, sample size, data collection, data measurement, and data analysis; **b] intervention characteristics**–intervention description, timeline, pedagogical approach [teacher-centered approach/student-centered approach] [15], communication synchronicity [synchronous/asynchronous] [16], and intervention delivery modes [desktop-based/web-based/TV-based/radio-based/paper-based modality] [19]; and **c] intervention outcomes**—socioemotional and behavioral changes such as mental health, social interaction, or academic-related behaviors, attitudes or perceptions, difficulties or burdens, and academic achievement. Data were tabulated to compare between each intervention characteristics such as teacher-centered and student-centered approach with and without synchronicity. All variables were analyzed in frequency and outcomes would be evaluated into positive, negative and mixed effects [qualitative approach] if there was a mix of statistical analyses used in each outcome. The missing results were not evaluated due to qualitative analysis of this review.

### Ethical considerations

The study was approved by the Institute for the Development of Human Research Protections, Thailand [IHRP 175/2564].

## Results

**Fig 1:**
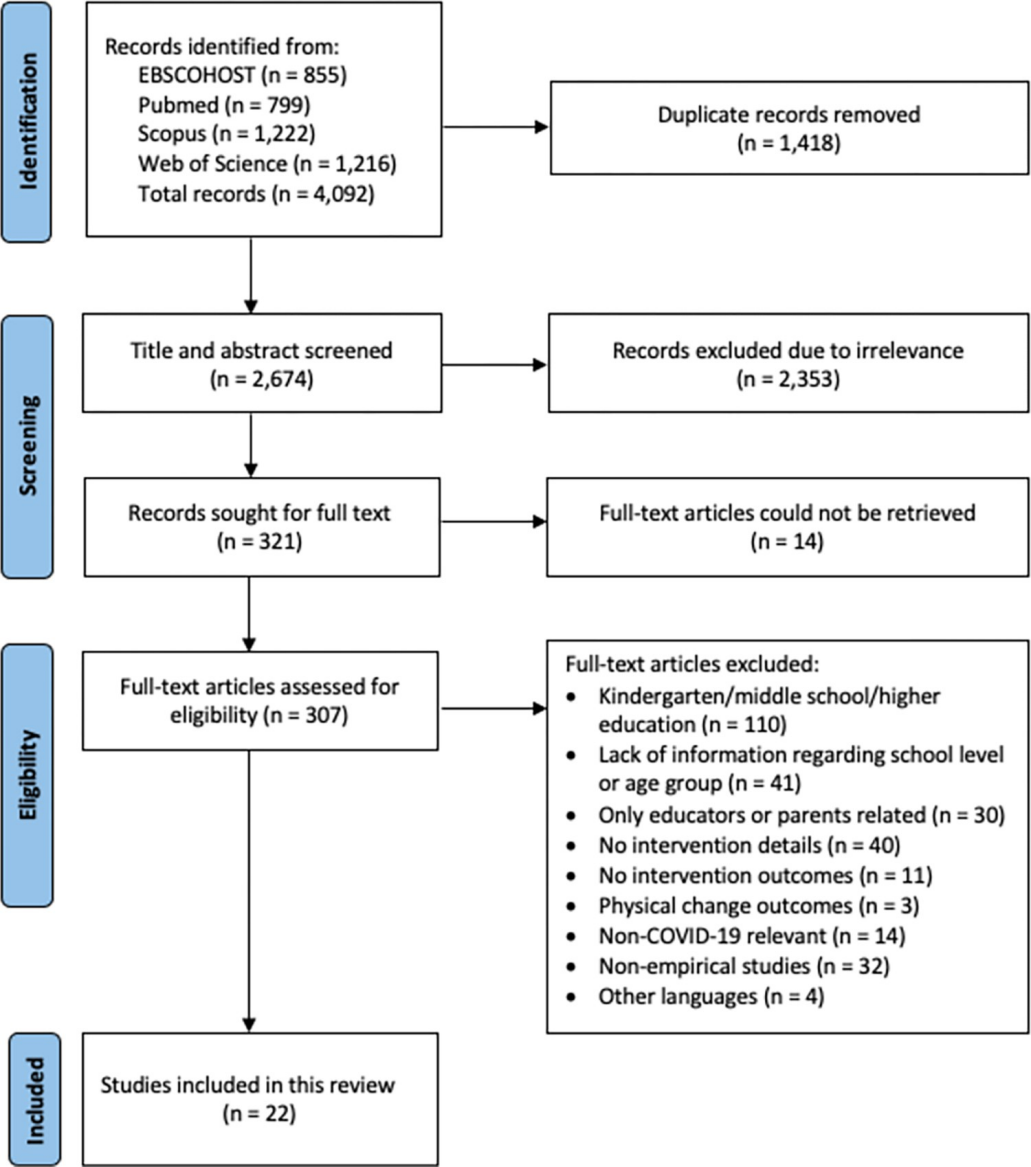
PRISMA flow diagram of the study selection process.

There was a total of 4,092 articles obtained from the four selected databases from which 1,418 duplications were removed, see Fig 1. During the screening process, 2,352 records were removed due to irrelevance and 14 records could not be retrieved in full text. Three hundred and eight full-text records were reviewed for eligibility, and some were excluded owing to the different or unidentifiable target groups and having no intervention details and outcomes in total of 285 articles. Finally, 22 studies met the eligibility criteria and were included for data analysis.

### Study characteristics

The characteristics of study covered study setting, type of study, target population, data collection and data analysis.

The majority of included studies [n = 11] were from Europe followed by Asian countries [n = 8], see Table 3. The remaining studies were scattered across different countries in American and Middle-Eastern regions. About two-thirds of the articles were interventional studies [n = 15] and all of them were quasi-experimental studies. Seven studies were non-interventional or observational studies consisting of observation analytic study [cross-sectional study] [n = 5] and descriptive study [qualitative] [n = 2]. The target groups of 11 studies were specifically primary school children in grade 4 to 6 or children aged around 10 to 12 years old, while participants in other included studies were mixed across early and late elementary school levels [n = 8] and only three studies related specifically to children in grades 1 to 3.

**Table 3:** Study characteristics of included studies.

Author	Title	Publication Year	Country	Objective	Study design	Methodology					
						Target group [age]	Settings	Sample size^α^	Data collection^α^	Data measurement^α^	Data analysis^α^
Beach KD et al. [20]	Pivoting an Elementary Summer Reading Intervention to a Virtual Context in Response to COVID-19: An Examination of Program Transformation and Outcomes	2021	United States of America [U.S.]	To examine the transformation of a traditional summer reading intervention [SRI] to synchronous virtual format in response to school closures due to COVID-19	Interventional study [Quasi-experimental study]	Second and third grade students/Educator/Caregivers	Title 1 primary schools [for low-income families]	35	Purposive sampling/ Before and after each set of 10 lessons taught	Daily attendance, Sound Partners mastery test, Oral reading fluency^β^	Mean, SD and paired-samples t-tests
Çetin H & Türkan A [21]	The Effect of Augmented Reality based applications [ARI] on achievement and attitude towards science course in distance education process	2021	Türkiye	To examine the effects of these applications on attitude and academic achievement towards the science course	Interventional study [Quasi-experimental study]	Third grade students [8 years old]	15 primary schools in city center of Siirt	15	Convenience sampling/ Before and after intervention	Questionnaire about Science achievement test [Electric Vehicles], Attitude scale for science course^β^	One sample t-test
Christopoulos A & Sprangers P [22]	Integration of educational technology during the COVID-19 pandemic: An analysis of teacher and student receptions	2021	Belgium	To analyze the integration of an educational technology platform and relate the difficulties of education that teachers and students faced amidst the COVID-19 pandemic	Interventional study [Quasi-experimental study]	Fifth to eighth students	14 regional primary schools	335 [30 primary school students]	Convenience sampling/After intervention	**Quantitative:** Questionnaire about knowledge-assessment tests and psychometric instrument **Qualitative:** Semi-structured interviews about experience	**Quantitative:** Percentage **Qualitative:** Thematic analysis
Cunha J et al. [23]	No Children Should Be Left Behind During COVID-19 Pandemic: Description, Potential Reach, and Participants’ Perspectives of a Project Through Radio and Letters to Promote Self-Regulatory Competences in Elementary School	2021	Portugal	To analyze potential reach of self-regulated learning [SRL] competences using alternative modes of intervention and explore participants’ perspectives about their experience during this project	Interventional study [Quasi-experimental study]	Third and fourth grade students [8–9 years old**]**	8 primary schools	42	Purposive sampling/After intervention	Semi-structured interview about experience during the two modes of delivery [radio and letters]	Content analysis
Fiş Erümit S [24]	The distance education process in K–12 schools during the pandemic period: evaluation of implementations in Türkiye from the student perspective	2020	Türkiye	To evaluate the distance education initiative implemented by Türkiye’s Ministry of National Education for K-12 schools during the COVID-19 pandemic [from March to July], from the students’ point of view	Descriptive study [Interview]	First to twelfth grade students	Primary school in Trabzon	4 primary school students, 4 lower and 4 upper secondary school students	Convenience and criterion sampling	Semi-structured interview about experiences during the learning process and the views on distance education	Content analysis
Gim N [25]	Development of life skills program for primary school students: Focus on entry programming	2021	South Korea	To develop a program through the Entry program that uses online learning to improve their life skills, given the need to increase contactless online classes due to COVID-19	Interventional study [Quasi-experimental study]	Fifth and sixth grade student	8 primary schools from four cities and four rural areas	360	Purposive Sampling/After intervention	Life Skills Scale for Sport^β^ such as time management and teamwork.	ANOVA [Scheffe’s post-hoc test]
Huertas-Abril CA [26]	Developing Speaking with 21st Century Digital Tools in the English as a Foreign Language Classroom: New Literacies and Oral Skills in Primary Education.	2021	Spain	To explore the potential of an online video discussion platform to develop new literacies and oral skills in English as a foreign language [EFL] for Spanish Primary Education students	Interventional study [Quasi-experimental study]	Third to Sixth grade students [8–11 years old]	Semi-public school in the south of the province of Córdoba	82	Purposive sampling	Questionnaire about the use of Flipgrid to develop new literacies and oral skills in EFL in Spain^β^	ANOVA and two sample t-test
Ilhan GO, Kaba G & Sin M [27]	Usage of Digital Comics in Distance Learning during COVID-19	2021	Türkiye	To reveal the effect of digital comics material on students’ academic success, and their views on distance education, course and digital comics	Interventional study [Quasi-experimental study]	Sixth grade students	Private school in Istanbul [urban]	**Quantitative:** 10 **Qualitative:** 5	Purposive/Before and after intervention	**Quantitative:** Achievement Test^β^ **Qualitative:** Semi-structured interview about how students evaluate distance education^β^	**Quantitative:** Kruskal Wallis test **Qualitative:** Content analysis
Kiili K et al. [28]	Flow experience and situational interest in game-based learning: Cousins or identical twins	2021	Finland	To examine the effectiveness of the scaffolding mechanism to confirm that the game experience of the participants was not harmed by poor alignment of challenge and skills	Interventional study [Quasi-experimental study]	Fifth-grade students	8 schools in Helsinki [urban]	52	Purposive sampling / Before and after intervention	In-game-based number line estimation performance, Math anxiety questionnaire, Flow Short Scale, Situational interest	Paired-samples t-test, Pearson’s and Spearman’s correlation and Forced-entry multiple regression analyses
Loukomies A & Juuti K [29]	Primary students’ experiences of remote learning during covid-19 school closures: A case study of Finland	2021	Finland	To examine how students experienced the remote learning period, how they evaluated their study days and what emotions they experienced	Descriptive study [Interview]	Fifth-grade students	Comprehensive school in Helsinki	23	Convenience sampling/After intervention	Experience Sampling Method [ESM] with instant video blogging [IVB] about students’ perceptions of the remote learning period	Content analysis
Meeter M. [30]	Primary school mathematics during the COVID-19 pandemic: No evidence of learning gaps in adaptive practicing results	2021	Netherlands	To investigate whether forms of computer-assisted learning mitigate the decrements in learning observed during the lockdown	Interventional study [Quasi-experimental study]	Second to sixth grade students	810 primary schools [urban]	53,656	Purposive sampling/weekly data collection in pre-lockdown, lockdown [14 March– 11 May 2020], and post- lockdown period	Mathematics achievement Attainment Practice in number of exercises finished in a week:	Linear mixed model [LMM] analysis with random intercepts [each grade, class, and school]
Panskyi T et al. [31]	The Comparative Estimation of Primary Students’ Programming Outcomes Based on Traditional and Distance Out-of-School Extracurricular Informatics Education in Electronics Courses during the Challenging COVID-19 Period	2021	Poland	To analyze primary school students’ perception of programming in traditional and distance out-of-school learning modes and the impact of emotions on educational outcomes	Interventional study [Quasi-experimental study]	Age 12–13 years old	Primary school	44	Convenience sampling/After intervention	Questionnaire about satisfaction, enjoyment, and motivation	Paired t-test
Park S & Kim S [32]	Is Sustainable Online Learning Possible with Gamification? -The Effect of Gamified Online Learning on Student Learning	2021	South Korea	To prove the causal relationship between online gamified content and learners’ motivation to learn	Interventional study [Quasi-experimental study]	Fifth grade student/Seventh grade student	Primary and secondary schools	85 fifth grade students and 55 seventh grade student**s**	Convenience sampling/after intervention	Science Level Up questionnaire^β^ about motivation to learn science, self-efficacy, self-determination, grade motivation and career motivation	Mean, SD, skewness, kurtosis, tolerance and variance inflation factors analysis, and ANOVA
Simpson JC [33]	Distance learning during the early stages of the COVID-19 pandemic: Examining K-12 students’ and parents’ experiences and perspectives	2020	United States of America	To explore how Pre K-12 students learn at a distance, what strategies and tools are most successful, and what challenges they face related to learning at home during the COVID-19 crisis	Observational analytic study [Cross-sectional study]	Fourth to twelfth grade students/Caregivers	Public, private and charter schools in Texas	155	Convenience sampling	**Quantitative& Qualitative:** Questionnaire and interview about online learning tools and strategies, video conferencing, online learning workload, access to WiFi and devices	**Quantitative:** Percentage **Qualitative:** Thematic analysis
Spitzer MWH & Musslick S [34]	Academic performance of K-12 students in an online-learning environment for mathematics increased during the shutdown of schools in wake of the COVID-19 pandemic	2021	Germany	To examine the impact of the school closures on the performance of K-12 students in an online learning environment for mathematics by contrasting students’ performance before the shutdown against their performance during the shutdown.	Interventional study [Quasi-experimental study]	Fourth to tenth grade students	Public and private schools	13,249	Purposive sampling/Before and during intervention	Data from software about a] which problem set was computed b] the number of distinct problems set that each student computed for a given book topic, and c] the number of times a student repeated a given problem set	LMM with fixed effects and random effects, and absolute error and relative error rate
Stalin LT & Kim Hua T [35]	Use of snapchat to enhance primary school English as second language learners [ESL] in the writing of personal information	2020	Malaysia	To measure to what extent Snapchat can enhance primary school ESL learners’ sentence construction in writing personal information	Interventional study [Quasi-experimental study]	Second grade student	National school in a semi-urban area in Johor	30	Stratified sampling/ Before and after intervention	The test from Common European Framework of References syllabus to write 3 simple sentences for each topic given	Paired t-test
Tajik F & Vahedi M [36]	Quarantine and education: an assessment of Iranian formal education during the COVID-19 outbreak and school closures	2021	Iran	To understand the general points of view/ effectiveness/efficiency of the most used social media sources/ MOOCs/educational television	Observational analytic study [Cross-sectional study]	First to twelfth students/Educators	N/A	593	Convenience and snowball sampling	Questionnaire about age, interaction, educational design, time management, motivation, learning actively, flexibility, and the visual learning environment^β^	**P**ercentage and Pearson and Spearman correlation
Wang D et al. [37]	Online Teaching during the "School Is Out, but Class Is On" Period: Based on 33,240 Online Questionnaire Surveys across China	2020	China	To understand the online teaching situation and the attitudes of different subjects towards online teaching during the “School is Out, but Class is On” period	Observational analytic study [Cross-sectional study]	First to twelfth grade student/ Educators/School administrators/Caregivers	District and country schools in 28 provinces	17,025	Convenience sampling	Questionnaire about attitude towards online learning	Percentage
Wang X et al. [38]	Understanding Learner Continuance Intention: A Comparison of Live Video Learning, Pre-Recorded Video Learning and Hybrid Video Learning in COVID-19 Pandemic	2021	China	To identifying the factors influencing students’ continuance intention toward online learning during the COVID-19 pandemic, and the differences in acceptance among three online video learning modes [live video, recorded video and hybrid video learning]	Interventional study [Quasi-experimental study]	Fourth to ninth grade students [age 9–15 years old]	Hubei Province	306,139	Convenience sampling/After intervention	Questionnaire about parental involvement in their children’s motivation and engagement, children’s academic work, supervision, and interactions	The structure equation modeling [24] to test hypotheses comparing the path coefficients
Wijaya TT [39]	How Chinese students learn mathematics during the coronavirus pandemic	2021	China	To show how students in China learn mathematics during the coronavirus pandemic by investigating students’ attitude towards Hawgent dynamic mathematics software’s learning video	Observational analytic study [Cross-sectional study]	First to twelfth grade students	N/A	408 [47.1% from primary school]	Purposive sampling	**Quantitative:** Questionnaire about the attitude towards Hawgent dynamic mathematics software^β^ **Qualitative:** Questions about opinions towards the advantages and disadvantages of using the learning video	**Quantitative:** Percentage **Qualitative:** N/A
Xie ZY et al. [40]	Micro classes as a primary school-level mathematics education response to COVID-19 pandemic in China: students’ degree of approval and perception of digital equity	2021	China	To explore the student’s degree of approval and perception of digital equity of micro classes for primary school–level mathematics in China during COVID-19 by tracking and investigating the NCPM [Chinese New Century Primary School Mathematics Textbook] micro class	Observational analytic study [Cross-sectional study]	First to sixth grade students [age 6–12 years old]	222 primary schools	132,740	Stratified sampling	Questionnaire about survey notes, personal background information, and degree of approval with the micro class^β^	Percentage
Yen ELY & Mohamad M [41]	Spelling mastery via google classroom among year 4 elementary school students during the covid-19 pandemic	2021	Malaysia	To determine the effectiveness of Google Classroom for mastering spelling among elementary school ESL students during the Movement Control Order [MCO] imposed by the COVID-19 pandemic	Interventional study [Quasi-experimental study]	Fourth grade student [age 10 years old]	National primary school in Selangor	30	Purposive sampling/Before and after intervention except field notes taken during intervention and interview taken after intervention only	**Quantitative:** The spelling questions on the word lists used in the English textbook^β^ **Qualitative:** Semi-structured interview about the feedback regarding the intervention and assisting mastery of spelling^β^ and Field notes	**Quantitative:** Number **Qualitative:** Triangulation of data

Note: ^α^related to primary school students only ^β^have been tested for validity or reliability

All the studies included applied non-randomization sampling, for example, convenience or purposive sampling for quasi-experimental and descriptive studies. For the cross-sectional studies, a mix of sampling techniques were used. Data measurement depended on study outcomes of interest, but most of the quantitative studies employed survey questionnaires as measurement tools, and the qualitative studies generally used semi-structured interviews. Data analysis methods depended on the objectives of each study; for example, the studies that aimed to identify association between variables applied Pearson’s and Spearman’s correlation analysis or t-test and ANOVA test of pre- and post-test analysis with and without control group for causal relationship evaluation.

### Intervention characteristics

A wide range of intervention periods was observed among the nine interventional studies, ranging from five days to five months [see Table 4]. Fifteen studies focused only on student-centered learning, five studies contained a combination of student-centered and teacher-centered approaches, and two studies utilized the teacher-centered method only. Communication between the teacher and pupils was grouped into three categories: synchronous [n = 9], asynchronous [n = 7], and a combination between the two [n = 6]. Desktop-based [n = 9] and web-based modalities [n = 7] were the most popular interventions, followed by a mixed modality [n = 5] and radio or paper-based modalities [n = 1].

**Table 4:** Intervention characteristics and outcomes of included studies.

Author	Intervention characteristics				Results				
	Description	Period [timeline]	Pedagogical approach/ Communication synchronicity	Intervention delivery modes	Academic performance	Academic-related behavior	Attitude/perception	Difficulty/ Burden	Mental health/ social interaction
Beach KD et al. [20]	SRI was a one-to-one instruction class.	22 days [July 2020]	Student-centered/ Synchronous	Web-based modality	The second and third grade students maintained scores on oral reading fluency [p-value; p = 0.93 & 0.79] and accuracy [p = 0.35 & 0.50], and increased by approximately 10.5 and 9.8 percentage points on the Sound Partners mastery test [p = 0.002; Cohen’s effect size: d = 1.30 & p < 0.001; d = 1.25]	Student attendance in the virtual SRI was approximately 89% of all instructional sessions.			
Çetin H & Türkan A [21]	ARI was an application of three-dimensional technology supporting individuals to understand and perceive the real world surrounded by objects created in a virtual environment.	4 weeks	Student-centered/ Synchronous	Desktop-based modality	The ARI statistically increased the achievement of students in science [t-value: t = -11.60; degrees of freedom: d = 14, p < 0.01].		The ARI statistically increased students’ attitudes towards science in terms of interest [t = -8.60, d = 14, p < 0.01], enjoyment [t = -2.185, d = 14, p < 0.05], and total score [t = -9.18, d = 14, p < 0.01].		
Gim N [25]	A life skill program is a free web-based platform including three conditions necessary for students’ flow: the educational goal of the day, the task to expand the scaffold, and feedback that could reflect improvement. It lasted 50 min per session.	8 weeks	Student-centered/ Synchronous	Desktop-based modality	It was confirmed that the 6-week and 8-week intervention [C&D] groups had higher life skill scores than the A and B groups [p < 0.01].	Scheffe’s post-hoc test results revealed that the higher the frequency of participation in life skills, the higher the life skills score [p < 0.01].			
Meeter M **[30]**	Snappet was installed on tablets for students to practice their mathematical skills. Students received immediate feedback on each exercise. Data from each student was collected on a real-time dashboard for the teacher to adjust their instructions or give personal feedback.	8 weeks [14 March to 11 May 2020]	Student-centered/ Synchronous	Desktop-based modality	During the period of school closures, students progressed more effectively in academic achievement when measured against themselves and their peers in previous years, and this was especially true for younger students [grades 4 and 5].	Students finished more exercises on Snappet during and after the lockdown in 2019–20 than their peers had done in 2018–19.			
Panskyi T et al. [31]	The traditional Arduino kits was a computer program for traditional/stationary out-of-school education during weekends while TinkerCad circuits was a MS team software application for distance education on both weekdays and weekends. There were 10 sessions and each session lasted 1.5 hours.	Tinkercard [4 months—March to June 2020] Arduino [4 months—December 2019 to March 2020]	Student-centered/ Synchronous	Web-based modality	The learning modes [distance or traditional] had no significant impact on students’ ability to learn the course materials and perform tasks.		Students’ enjoyment, satisfaction, and motivation revealed a significant difference in favor of the traditional learning mode.		
Stalin LT & Kim Hua T [35]	Snapchat was an application used to talk with friends, share photos, videos, and play around with filters. The pictures uploaded in Snapchat are deleted automatically after 24 hours. It is used to attract participants’ attention and interests to enhance their sentence construction skills in writing personal information.	5 days	Student-centered/ Synchronous	Web-based modality	The test revealed a significant difference in students’ scores between pre and post-writing tests [t[29] = −18.997, p < 0.001] with a positive increase in the mean score of pre- and post-writing tests.				
Yen ELY & Mohamad M [41]	Google classroom was used as the online learning platform for teaching students how to spell. In each lesson, ten pictures with audio pronunciations were uploaded for discussion. Participants would then access a Google Form via Google Classroom to complete the spelling quizzes. This activity was repeated for five consecutive weeks.	5 weeks	Student-centered/ Synchronous	Web-based modality	Participants in this intervention experienced a more significant improvement for achieving better spelling scores compared to those who did not participate.	The use of Google Classroom revealed an improvement and increased motivation among users in mastering spelling from the perspective of active participation and teamwork.			
Huertas-Abril CA [26]	Flipgrid was an online free video discussion platform that allows educators to create “grids” to host and facilitate video discussions, which can hold an unlimited number of topics and responses.	N/A	Student-centered/ Synchronous	Web-based modality			90.3% of students showed a positive attitude, which was also reflected in the use of Flipgrid [84.1%].	There was a balance between those who had problems using the digital tool [45.1%] and those who could use it with no major difficulties [54.9%].	
Kiili K et al. [28]	The math game research environment allowed for the creation of web-based math games that can include a variety of different kind of number line-based tasks and instructional features; it also supports collection of both behavioral and self-reported data. Teachers were held five lessons [45 minutes each] within a four-week period in which students would complete the pre-test, play the Number Trace game, and complete the post-test.	4 weeks [Spring 2020]	Student-centered/ Synchronous	Desktop-based modality	Students performed very well as the mean estimation accuracy [e.g. game performance] was 93.9%. Accuracy was significantly higher in the post-test [mean = 92.1, SD = 3.15] than in the pre-test [mean = 84.3, SD = 7.18, t[51] = 8.75, p < 0.001, d = 1.21].				
Christopoulos A & Sprangers P [22]	The Intelligent Tutoring Systems [ITS] and Learning Analytics [LA] platform was used to provide fully customized learning paths with multiple lessons and exercises. Real-time data was used to adapt the lessons based on student performance.	12 weeks	Student-centered/ Asynchronous	Desktop-based modality	Students indicated that there was "moderate" impact from the ITS platform on their learning through learning repetition and creativity exercises.	Exercises impacted students’ motivation differently. For example, some appreciated the intervention and wanted to keep practicing, whereas others felt frustration and dissatisfaction.	The majority of primary and secondary school students voted "good" for their experiences with the ITS. Primary school students positively interpreted the gamified elements [trophies, reward system, competition dashboard] due to pleasance.	Students pointed to occasional technical issues [e.g. computers freezing, platform crashes, inadequate response times] or the inability of the school infrastructure to support the platform’s operational requirements.	
Ilhan GO, Kaba G & Sin M [27]	An 80-minute-Social Studies Course on the Zoom application was conducted once a week. It was carried out through digital comic materials in addition to textbooks.	3 weeks [academic year 2019–2020]	Student-centered/ Asynchronous	Web-based modality	The digital comic books positively affected academic achievement [median pre-test = 8, post-test = 14.5] and the difference was statistically significant [t = 36, z = -2.52, p = 0.012].		Almost all participants had negative opinions about distance education, particularly the communication style and home learning environment.	Students experienced problems with connected devices such as computers, tablets, smart phones, etc. in terms of screens freezing or general network infrastructure problems.	
Park S & Kim S [32]	Science Level Up was an online learning gamification tool used for teaching science. Instructors created an instructors-only account and registered learners/students. This enabled instructors to monitor the progress of learners by providing information regarding the programs that have been completed and how many levels learners had earned.	8 weeks	Student-centered/ Asynchronous	Desktop-based modality	Science Level Up had a positive impact on learners’ understanding of educational content due to enjoyment [SD = 0.69, t = 6.02, p < 0.01].	Science Level Up had a positive impact on learners’ motivation [SD = 0.52, t = 4.88, p < 0.01], self-efficacy [SD = 0.58, t = 4.27, p < 0.01], self-determination [SD = 0.58, t = 4.27, p < 0.01], career motivation 0.43 [t = 2.84, p < 0.05], and grade motivation [SD = 0.55, t = 2.7, p < 0.05].			
Spitzer MWH & Musslick S [34]	The Bettermarks software covered the mathematics curricula in Germany from classes 4–10 and contained 100 mathematics topics. Teachers initially assigned problem sets to students. If students received negative feedback on their first attempt of a problem within a problem set, they could attempt that problem again.	Before and during 15 March 2019 to 15 June 2019	Student-centered/ Asynchronous	Desktop-based modality	Students’ performance improved during the shutdown of schools relative to the year before. The absolute error rate of students during the shutdown was significantly lower than before the shutdown [b = -2.37e-02; t = -8.39; p < .001]. The relative error rate also saw a significant decrease by 2.43% during the school shutdown compared to the same time in the previous year [b = -1.21e-02; t = -5.06; p < .001].				
Wijaya TT [39]	Hawgent dynamic mathematics software was a problem-based learning approach with virtual teachers. The learning videos discussed problems faced in daily life using mathematics.	N/A	Student-centered/ Asynchronous	Desktop-based modality		Students stated that they could arrange their own study time and could concentrate better.	Almost all students [93.66%] enjoyed learning using Hawgent dynamic mathematics software videos and they did not feel that video learning during the coronavirus pandemic was boring.		
Xie ZY et al. [40]	Micro class was an online videos course. It consisted of an introduction, teaching and learning interaction, and summary and consolidation. In the introduction, teachers introduced the purpose and goals of the class and then proceeded to conduct interactive activities with students. Finally, a summary and practice session concluded the course.	Since mid-February 2020	Student-centered /Asynchronous	Desktop-based modality			Micro classes achieved a high degree of approval by students during the pandemic period [90%].		
Tajik F & Vahedi M [36]	Television program was not an interactive program in Iran. Social media was categorized into social networking and Web 2.0. MOOC was an interactive learning platform associated with the international sites of edX and Coursera.	3 months	Teacher-centered/ Asynchronous [TV, social media, and MOOC]	Televisual-based modality [TV]/Web-based modality [social media and MOOC]		Television content motivated students more than MOOCs and social media.			
Wang X et al. [38]	A live video was considered as one of the tools for a synchronous course via cable television or online learning platforms. The pre-recorded video was utilized in an asynchronous online class and was prepared and recorded in video format prior to students beginning their learning.	1 week [2–9 May 2021]	Teacher-centered/ Synchronous [live video]	Web-based modality [live video]			The most preferable method of video learning was the use of a combination of live conference and pre-recorded video learning. This method received the highest scores in terms of interaction, engagement, satisfaction, perceived usefulness, and continuous attention.		Parental involvement on interaction showed the highest value when using a live video learning. More parental involvement resulted in better online learning outcomes.
			Teacher-centered/ Asynchronous [pre-recorded video]	Televisual-based modality/Web-based modality [pre-recorded video]					
Fiş Erümit S [24]	The Education Information Network [EBA] content portal was an online learning platform that allowed students and teachers to log into synchronous lessons. It also enabled teachers to send homework to students. EBA TV channels were broadcast via the EBA content portal from 09:00 to 14:00 and then replayed between 14:30 and 19:30.	4 months [March to July 2022]	Student-centered /Synchronous [EBA portal]	Desktop-based modality [EBA portal]		The engagement varied depending on students’ personal preferences. Students were concerned whether the teachers could motivate students to learn [EBA portal].	Some students were ambivalent and wished for a hybrid education system. The EBA portal was easily accessible. Students were concerned with how much they could learn from teachers’ instructions [EBA portal].		Students reported that the synchronous lessons gave them the opportunity to interact with their friends/teachers as a positive outcome [EBA portal]. However, it required family support and needed more opportunities to communicate with teachers.
			Teacher-centered/ Asynchronous [EBA TV]	TV-based modality [EBA TV]		Students’ engagement with topics varied depending on their personal needs or preferences. Some students noted that engagement stemmed from greater responsibility and individual effort compared to face-to-face education [EBA TV].		Students stated that teachers poorly managed students’ difficulties during synchronous lessons and did not utilize technological devices other than the blackboard [EBA TV].	
Cunha J et al. [23]	Yellow Trials and Tribulations was a radio program about the adventures of the colors of the rainbow searching for their friend. It was broadcast every Tuesday from 19:10 to 19:30, and paper activities were sent to participants through mail or via personal delivery.	6 weeks [radio] and 12 weeks [letters]	Teacher-centered/ Synchronous [radio]	Radio-based modality [radio]			Children considered the radio intervention very creative [radio].	Children reported facing heavy school workloads, which prevented them from completing the activities [radio & letters].	Children reported that they were happy their parents could listen to the story with them and help them with suggestions to complete the activities. They mentioned the absence of teacher support to help them complete school tasks [radio].
			Student-centered/ Asynchronous [letters]	Paper-based modality [letters]			Children enthusiastically stated that the letters helped them learn how to think through fun and creative activities [letters].		
Simpson JC [33]	Multiple learning strategies were used to educate students via distance learning such as 1] live video conferencing [Zoom, Google Meet, Microsoft Teams, etc.], 2] teacher-created instructional videos, other instructional videos [YouTube, BrainPOP, etc.], 3] adaptive learning programs [IXL, Achieve 3000, Istation, etc.] and game-based tools [Nearpod, Quizizz, etc.], 4] communication and discussions programs [Fligprid, Padlet, etc.], and 5] eBooks, digital worksheets, and assignments.	Spring 2020	Mixed/Mixed	Web-based modality		Game-based learning was often mentioned as a successful distance learning engagement strategy.	Preferred strategies included live video conferencing [77%] and teachers recording their own instructional videos [72%]. Factors which resulted in unsuccessful strategies consisted of lengthy videos, classroom management issues and security concerns, and lack of organization and standardization. They also recommended increasing teacher-guided instructions, reducing workload and assignments, and improving and streamlining communications.	Students reported challenges in accessing the technology.	Students reported the lack of opportunities for socialization and high screen time.
Wang D et al. [37]	Four commonly used online teaching and service modes were applied: TV teaching videos [broadcasted live through TV stations or dedicated digital TV channels], live classes through an online platform, interactive online classes with online tutoring and Q&A sessions, and optional resources and online guides [independent study through websites, class exchange groups, and teachers answering questions through QQ and WeChat].	1 week [5–11 February 2020]	Mixed/Mixed	Televisual-based modality/Web-based modality/ Desktop-based modality		Students had insufficient online self-learning ability and it was difficult to guarantee learning participation. The results showed that 38.5% of students had low confidence in adapting to online learning quickly; 37.1% believed that they were less active in online learning.	The results showed that although 17.0% of students expressed some concerns, 83.1% still had a positive attitude [36.9% for “surprised” and 46.1% for “happy”]. This was mainly because students had a positive understanding of online learning.		45.8% said that teachers and parents were required to supervise students to complete the online learning tasks.
Loukomies A & Juuti K [29]	Digital Learning was conducted via an MS Teams meeting for 30 minutes and consisted of a teaching session and instructions for individual tasks that started at 10:00, followed by discussion about topics for the afternoon session. By 14:00, students had to submit their assignments to a Teams folder. Teachers were on standby in case students wanted to call or ask questions.	8 weeks [18 March to 13 May 2020]	Mixed/Mixed	Web-based modality			Among 369 video records, students mentioned positive feelings 871 times and negative feelings 296 times. Examples of these feelings consisted of boredom [25 times], irritation [20 times], difficult tasks [75 times], and unable to learn anything [41 times]. Positive learning-related emotions was mentioned 225 times, which comprised variations of nice or good.		

Note: Green boxes indicate only positive outcomes; yellow boxes indicate mixed outcomes ranging from positive to negative; and red boxes indicate only negative outcomes

In summary, the characteristics of remote or distance education were classified into a synchronously student-centered approach [n = 9], an asynchronously student-centered approach [n = 6], mixed approaches between student-centered and teacher-centered approaches regardless of synchronicity [n = 7], and a synchronously or asynchronously teacher-centered approach [n = 1], respectively. Examples of the synchronously student-centered approach consisted of exercises or discussions where students received real-time feedback through Snappet and Google classroom [30, 41]. The asynchronously student-centered approach was presented via gamification or exercises where students were given flexible learning times such as the Science Level Up program [32]. On the other hand, examples of synchronously and asynchronously teacher-centered approaches comprised lessons that were taught via live video, recorded video, or radio broadcasting [23, 38].

### Intervention outcomes

Several outcomes of interventions supporting remote or distance education among primary school children during the COVID-19 pandemic were assessed in terms of attitudes/perceptions [n = 13], academic performance [n = 12], academic-related behaviors [n = 11], difficulties/burdens [n = 6], and mental health/social interaction [n = 5]. Each study has one or more than one of these respective assessments.

Positive effects on academic performance were found in any educational type of intervention compared to those who did not receive interventions or before exposure to interventions. Academic-related behavior outcomes such as engagement or motivation had positive effects in synchronously student-centered interventions [20, 25, 30, 41], while other interventions produced a mix of positive and negative results. Difficulties with remote or distance education in all studies were mostly associated with technical issues such as screens freezing or the inability of the school’s infrastructure to support the platform’s operations [22, 23, 26, 27]. On the other hand, different attitudes or perceptions toward remote or distance education was seen across all intervention types depending on the individual student; for example, some students felt bored and concerned while others perceived enjoyment [29, 36]. A similar situation was found for mental health or social interaction where students appreciated the opportunity to interact families, friends, and teachers but also required a support system from parents and teachers to do so [23, 24, 37, 38].

### Quality assessment

The JBI critical appraisal tools for analytical quasi-experimental studies [n = 15], cross-sectional studies [n = 5], and qualitative studies [n = 2] were applied to all included articles. Results are shown in a S1 File.

Most of the studies had an overall quality assessment score of higher than 50% [n = 19]; only three studies had a score of lower than 50% and all of them were cross-sectional studies. Among the cross-sectional studies [n = 5], most did not address confounding bias or apply strategies to deal with confounders [33, 36–39]. In addition, some studies did not clearly define inclusion and exclusion criteria and settings as well as valid and reliable measurements of exposure; however, these defined valid and reliable measurements of outcomes and appropriate analytical methods [36, 38, 40]. Among fifteen quasi-experimental studies, almost all studies clearly mentioned cause-and-effect variables, similarity between comparisons, exposure of similar treatment between comparisons, outcomes of comparisons measured in the same way, and appropriate data analysis methods. Nevertheless, various limitations among some studies were seen such as comparisons with the control group, evaluation of pre- and post-exposure, complete follow-up, and reliable outcome measurements.

## Discussions

The discussion of this study aligned with the results, which were classified into four parts: study characteristics, intervention characteristics, intervention outcomes, and study limitations and recommendations.

### Study characteristics

This review shows that the included studies were conducted in diverse regions and countries including both high- and middle-income countries. A previous systematic review of emergency remote education for K-12 by Crompton et al. [2021] noted that the COVID-19 pandemic was different from other emergency situations because it had a global reach and significantly impacted low-income countries [6]. Accordingly, further research in other low-income regions such as the Middle East, South Asia, South-East Asia and African is needed.

We find that the target group of most remote or distance education studies is less likely to be elementary school students. In this study, the majority of participants included were late primary school students. Therefore, the findings highlight gaps in which further research is needed especially among young children during early primary school year. This will help determine how significant the impact of remote or distance education is on the development of learning and skills as younger students require in-person learning the most [42].

### Intervention characteristics

There are various pedagogical approaches to remote or distance intervention in the included literature. The most popular method is synchronous learning, followed by blended synchronicity. A study by Meeter [2021] and Yen and Mohamad [2021] showed that the synchronous student-centered interventions were mostly in the form of exercises or discussions where students received real-time feedback [30, 41]. According to Media Naturalness Theory, the level of synchronicity and social communication cues such as facial expression or body language determine the naturalness of media [43]. Therefore, the more synchronous and natural the learning methods, the more preferable they are. It is similar to the result of a study by Seraj et al. [2022], which found that although teachers are in favor of synchronous methods [44], a combination of synchronous and asynchronous methods is also addressed as an optimal approach for teachers’ and students’ flexibility [45]. Both methods have their own benefits and challenges, so they can be selected based on the different contexts and preferences of teachers and learners.

Online and multimodal learning strategies are preferable by many countries for national distance education policies. According to a study conducted by the Global Education and Technology Team, Education Global Practice, World Bank Group, the policy of remote learning preferred multimodal delivery systems over unimodal delivery systems [46]. They suggested that it is effective to increase coverage, but a clear communication strategy is needed to respond to the local needs and contexts [46]. Accordingly, the multimodal delivery system should be supported to ensure accessibility based on technology capacity and learning preferences.

### Intervention outcomes

For the outcomes of distance education interventions, academic performance was not affected by remote or distance education in any educational approaches, which means that remote or distance education is just as effective in terms of academic development as in-person learning. A study by Meeter [2021] among 53,656 students in 2^nd^ to 6^th^ grade from Netherlands showed that the average of learning achievement was stronger during the lockdown year compared to the year before, and it remained even the lockdown ended [30].

This idea is supported by a systematic review about the effectiveness of distance learning before the COVID-19 pandemic, which showed that distance education is as effective as face-to-face learning in terms of student learning outcomes [74% of literature in the systematic review] [47]. Although a meta-analysis study by Ulum [2022] revealed that online education during the COVID-19 pandemic had moderate effects on academic performance compared to traditional learning, it has not been influenced by different online education approaches [48]. Therefore, in terms of academic outcomes, any pedagogical approaches and synchronicity in remote or distance education can be applied during public a health emergency.

Academic-related behavior outcomes, attitudes, and perceptions are determined by various learning approaches. The synchronously student-centered interventions are more engaging compared to other types. A study by Yen and Mohamad [2021] addressed that the use of Google Classroom revealed an increase in motivation among users in mastering spelling from the perspective of active participation and teamwork [41]. Compared to a study by Christopoulos and Sprangers [2021] about asynchronously student-centered approach that some students appreciated the intervention and wanted to keep practicing, whereas others felt frustration and dissatisfaction [22]. According to a study by Aguilar et al. [2022], there is a substantial association between live instruction and student engagement in online learning among primary school pupils in California [49], making synchronized student-centered interventions more engaging than other types. There was a 26% increase in the likelihood that students will finish all of their assignments for every additional hour of live instruction per week [49]. Student engagement can be explained by self-determination theory consisting of autonomy [feel in control of our own behaviors and goal], competence [feel competent and effective], and relatedness [experience interaction and feel connected] [50]. A synchronously student-centered approach can easily achieve these factors, especially for relatedness and competence, while an asynchronous approach encourages autonomy. Therefore, different learning approaches may not fully determine academic-related behavior outcomes but addressing each student’s self-determination and personal preferences are more important.

Remote and distance education intervention success depends on organizational factors such as technology infrastructure readiness, personal factors such as familiarity with technology and family support, and pedagogical factors such as course design and course delivery [51]. All these factors are addressed by the included studies in this review and there are similar views towards difficulties and burdens, and mental health or social interaction. Students mostly complained about technological problems and requested for support from families and teachers. It is similar to qualitative studies from parents and educators about the impact of remote learning on primary students’ well-being in that it has both positive and negative effects determined by supports from teachers, parents, and schools [52]. This emphasizes the importance of technology infrastructure preparation and a solid support system for learners.

### Quality assessment

Most of the study are quasi-experimental study and there are various limitations in comparisons with the control group, evaluation of pre- and post-exposure, complete follow-up, and reliable outcome measurements. A study by Huertas-Abril [2021] showed limitations in comparisons with the control group or pre- and post-exposure evaluation, and incomplete follow-up [26]. Quasi-experimental study is a manipulation of intervention to study group with non-equivalent control and quasi-independent variables [53]. Quasi-experimental study needs control or comparison group which can be one group design of pretest and posttest or non-equivalent control design [53]. According to experimental design, the follow up data is necessary to interpret the results, so it is necessary to have strategy dealing with incomplete follow-up or selection bias such as describing characteristics of loss follow-up group [54]. Thus, future research should reduce these biases to improve research quality in this field.

This review is a novel study that aims to systematically evaluate the outcomes of specific types of distance education interventions in primary school students who are significantly affected by education disruption compared to students at the secondary and tertiary levels. Nevertheless, this study also has several limitations. Firstly, there were multiple quasi-experimental studies in this review. Although this type of study can evaluate the causal relationship between intervention causes and effects, the issue of selection bias remains compared to randomized controlled trial studies [55, 56]. Furthermore, future quasi-experimental studies should concentrate on control group comparison and loss follow-up strategies. Secondly, as all studies were conducted during the COVID-19 pandemic, and it seemed difficult to collect the data for pre-tests, or to find appropriate control groups in the quasi-experimental studies. Therefore, continuous data monitoring on repeated measurements or a time series analysis will be of great value to better understand changes for the further program’s implementation. Lastly, the databases used may not have covered databases specific to the educational field since this might lead to selection bias. Future research should focus more on randomized study designs or should implement control groups for evaluation study and expanding database coverage for systematic review.

This study recommends that remote or distance education can be an alternative in primary school education to maintain academic performance during the crisis situation. Synchronously student-centered interventions are supposed to be promoted due to academic engagement. However, the technology infrastructure and support system from teachers and families should be considered by school administrators and policy makers to ensure effective remote or distance education.

## Conclusion

School closures due to the COVID-19 pandemic significantly disrupted the educational system and required the transition from in-person learning to remote or distance education. Consequently, different educational interventions were applied in various settings. In this study, the outcomes of each intervention were explored in primary school students. Most of the interventions were synchronously student-centered approaches utilizing online channels. In evaluating academic performance, remote or distance education was found to be effective in terms of academic development by using any learning approach, but the findings on attitudes and perceptions were mixed between positive and negative views. Positive academic-related behaviors were also seen when using a synchronously student-centered approach [e.g., positive outcomes in engagement]. Finally, difficulties or burdens, and mental health or social interaction reported similar results for all learning approaches. These included technological problems and support systems from families and teachers. While a synchronously student-centered method is recommended as the main intervention due to its excellent outcomes for academic achievement and engagement in academic behavior, other approaches can also be used in conjunction if it addresses students’ needs. Finally, the educational technology infrastructure and support system from teachers and parents are also necessary in remote or distance education. Future studies should further explore intervention outcomes on primary school students, especially in low-income regions, with a randomized study design.

## Supporting information

S1 File The JBI appraisal checklist of quasi-experimental, cross-sectional, cohort and qualitative studies. The protocol, template data collection forms, and data extracted from included studies were not publicly available and had not been registered.
(DOCX)

S2 File (DOCX)
