# Supplementary material for: Characteristics of distance education interventions and related outcomes in primary school children during COVID-19 pandemic: A systematic review
Source: PLoS One. 2023 Oct 13;18(10):e0286674. doi: 10.1371/journal.pone.0286674 (PMC10575523; doi:10.1371/journal.pone.0286674)
Supplement: S1 File — The protocol, template data collection forms, and data extracted from included studies were not publicly available and had not been registered. (DOCX) [file pone.0286674.s001.docx]

**Supplementary file**

**Table A Search term from each database**

| **Database** | **Search term** |
| --- | --- |
| Pubmed | (((((((((((("COVID-19"[Mesh]) OR (covid*[Title/Abstract])) OR (corona*[Title/Abstract])) OR ("SAR-Cov*"[Title/Abstract])) OR (CoV[Title/Abstract])) OR ("2019-nCoV"[Title/Abstract])) OR ("n-CoV"[Title/Abstract]))) AND ("elementary school"[Title/Abstract] OR "middle primary"[Title/Abstract] OR "upper primary"[Title/Abstract] OR "senior school"[Title/Abstract] OR "junior primary"[Title/Abstract] OR "primary school"[Title/Abstract] OR "student*"[Title/Abstract] OR "pupil*"[Title/Abstract] OR "child*"[Title/Abstract] OR "kid*"[Title/Abstract] OR "students"[MeSH Terms] AND ((journalarticle[Filter]) AND (english[Filter])))) AND (((((((((((((((distance education[MeSH Terms]) OR ("distance education"[Title/Abstract])) OR ("distance learning"[Title/Abstract])) OR ("online education"[Title/Abstract])) OR ("online learning"[Title/Abstract]))OR ("remote learning"[Title/Abstract])) OR ("remote education"[Title/Abstract])) OR ("virtual learning"[Title/Abstract])) OR ("virtual education"[Title/Abstract])) OR ("remote schooling"[Title/Abstract]))))) NOT (((((((((((((((((((((((((((((((((((universit*[Title]) OR ("higher education"[Title])) OR (postgrad*[Title])) OR (undergrad*[Title])) OR ("tertiary education"[Title])) OR (college[Title])) OR (campus*[Title])) OR (dent*[Title])) OR (nurs*[Title])) OR (pharmac*[Title])) OR (medic*[Title])) OR ("health professional*"[Title])) OR ("healthcare worker*"[Title])) OR ("health personnel"[Title])) OR ("health worker*"[Title])) OR (nonpharmaceutica*[Title])) OR (energy[Title])) OR (pharmaceutica*[Title])) OR (pharmacy[Title])) OR (clinic*[Title])) OR (pathology[Title])) OR (telemedicine[Title])) OR (inflammation[Title])) OR (patient*[Title])) OR (neurolog*[Title])) OR (telehealth[Title])) OR (surgery[Title])) ) OR (vaccin*[Title])) OR (antib*[Title])) OR (disorder*[Title])) OR (disabilit*[Title])) OR ("physical activit*"[Title])) OR (nutrition[Title])) OR (diet*[Title])) Filters: Journal Article, English, from 2020 - 2021 |
| Scopus | ( TITLE-ABS-KEY ( covid* ) OR TITLE-ABS-KEY ( corona* ) OR TITLE-ABS-KEY ( sar-cov* ) OR TITLE-ABS-KEY ( cov* ) OR TITLE-ABS-KEY ( 2019-ncov ) OR TITLE-ABS-KEY ( n-cov ) ) AND ( TITLE-ABS-KEY ( "elementary school" ) OR TITLE-ABS-KEY ( "middle primary" ) OR TITLE-ABS-KEY ( "upper primary" ) OR TITLE-ABS-KEY ( "primary school" ) OR TITLE-ABS-KEY ( student* ) OR TITLE-ABS-KEY ( pupil* ) OR TITLE-ABS-KEY ( child* ) OR TITLE-ABS-KEY ( kid* ) ) AND ( TITLE-ABS-KEY ( distance AND education ) OR TITLE-ABS-KEY ( distance AND learning ) OR TITLE-ABS-KEY ( online AND education ) OR TITLE-ABS-KEY ( online AND learning ) OR TITLE-ABS-KEY ( remote AND learning ) OR TITLE-ABS-KEY ( remote AND education ) OR TITLE-ABS-KEY ( "virtual learning" ) OR TITLE-ABS-KEY ( "virtual education" ) OR TITLE-ABS-KEY ( "remote schooling" ) ) AND NOT ( TITLE-ABS-KEY ( universit* ) OR TITLE-ABS-KEY ( "higher education" ) OR TITLE-ABS-KEY ( postgrad* ) OR TITLE-ABS-KEY ( undergrad* ) OR TITLE-ABS-KEY ( "tertiary education" ) OR TITLE-ABS-KEY ( college ) OR TITLE-ABS-KEY ( campus* ) OR TITLE-ABS-KEY ( dent* ) OR TITLE-ABS-KEY ( nurs* ) OR TITLE-ABS-KEY ( pharmac* ) OR TITLE-ABS-KEY ( medic* ) OR TITLE-ABS-KEY ( "health professional*" ) OR TITLE-ABS-KEY ( "health worker*" ) OR TITLE-ABS-KEY ( "healthcare worker*" ) OR TITLE-ABS-KEY ( "health personnel" ) OR TITLE-ABS-KEY ( nonpharmaceutica ) OR TITLE-ABS-KEY ( energy ) OR TITLE-ABS-KEY ( pharmaceutical ) OR TITLE-ABS-KEY ( pharmacy ) OR TITLE-ABS-KEY ( clinic* ) OR TITLE-ABS-KEY ( pathology ) OR TITLE-ABS-KEY ( telemedicine ) OR TITLE-ABS-KEY ( inflammation ) OR TITLE-ABS-KEY ( patient* ) OR TITLE-ABS-KEY ( neurolog* ) OR TITLE-ABS-KEY ( telehealth ) OR TITLE-ABS-KEY ( surgery ) OR TITLE-ABS-KEY ( vaccin* ) OR TITLE-ABS-KEY ( antib* ) OR TITLE-ABS-KEY ( disorder* ) OR TITLE-ABS-KEY ( disabilit* ) OR TITLE-ABS-KEY ( physical AND activit* ) OR TITLE-ABS-KEY ( nutrition ) OR TITLE-ABS-KEY ( diet* ) ) AND ( LIMIT-TO ( PUBSTAGE , "final" ) ) AND ( LIMIT-TO ( PUBYEAR , 2021 ) OR LIMIT-TO ( PUBYEAR , 2020 ) ) AND ( LIMIT-TO ( DOCTYPE , "ar" ) ) AND ( LIMIT-TO ( LANGUAGE , "English" ) ) |
| Web of Science | ((((((TS=(covid*)) OR TS=(corona*)) OR TS=(SAR-Cov*)) OR TS=(CoV*)) OR TS=(2019-nCoV)) OR TS=(n-CoV)) AND ((((((((TS=(“elementary school”)) OR TS=(“middle primary”)) OR TS=(“upper primary” )) OR TS=(“primary school” )) OR TS=(student*)) OR TS=(pupil*)) OR TS=(child*)) OR TS=(kid*)) AND ((((((((TS=(distance education)) OR TS=(distance learning)) OR TS=(online education)) OR TS=(online learning)) OR TS=(remote learning)) OR TS=(remote education)) OR TS=(“virtual learning”)) OR TS=(“virtual education” )) OR TS=(“remote schooling”) NOT (((((((((((((((((((((((((((((((((TS=(universit*)) OR TS=(“higher education”)) OR TS=(postgrad*)) OR TS=(undergrad*)) OR TS=(“tertiary education”)) OR TS=(college)) OR TS=(campus*)) OR TS=(dent*)) OR TS=(nurs*)) OR TS=(pharmac*)) OR TS=(medic*)) OR TS=("health professional*" )) OR TS=("health worker*")) OR TS=("healthcare worker*")) OR TS=("health personnel")) OR TS=(nonpharmaceutica)) OR TS=(energy)) OR TS=(pharmaceutical)) OR TS=(pharmacy)) OR TS=(clinic*)) OR TS=(pathology)) OR TS=(telemedicine)) OR TS=(inflammation)) OR TS=(patient*)) OR TS=(neurolog*)) OR TS=(telehealth)) OR TS=(surgery)) OR TS=(vaccin*)) OR TS=(antib*)) OR TS=(disorder*)) OR TS=(disabilit*)) OR TS=("physical activit*")) OR TS=(nutrition)) OR TS=(diet*) AND 2021 or 2020 (Publication Years) and Articles (Document Types) and English (Languages) |
| EBSCOHOST | TI ( covid* OR corona* OR SAR-Cov* OR CoV* OR 2019-nCoV OR n-CoV ) OR AB ( covid* OR corona* OR SAR-Cov* OR CoV* OR 2019-nCoV OR n-CoV ) OR ( DE "COVID-19" OR DE "POST-acute COVID-19 syndrome" ) AND TI ( “elementary school” OR “middle primary” OR “upper primary” OR “primary school” OR student* OR pupil* OR child* OR kid* ) OR AB ( “elementary school” OR “middle primary” OR “upper primary” OR “primary school” OR student* OR pupil* OR child* OR kid* ) OR ( DE "CHILDREN" OR DE "SCHOOL children") OR ( DE "PRIMARY schools") OR (DE "PLAY schools" ) OR ( DE "PRIMARY schools" OR DE "NATIONAL schools (Ireland)" ) AND TI ( “distance education” OR “distance learning” OR “online education” OR “online learning” OR “remote learning” OR “remote education” OR “virtual learning” OR “virtual education” OR “remote schooling” ) OR AB ( “distance education” OR “distance learning” OR “online education” OR “online learning” OR “remote learning” OR “remote education” OR “virtual learning” OR “virtual education” OR “remote schooling” ) OR ( DE "DISTANCE education" OR DE "EDUCATION" OR DE "SCHOOL administration" OR DE "COPYRIGHT & distance education" OR DE "TELEPHONE in education" OR DE "TELEVISION in education" OR DE "BLENDED learning" OR DE "COMPUTER assisted instruction" OR DE "CONTINUING education" OR DE "COURSEWARE" OR DE "CYBERSCHOOLS" OR DE "DISTANCE education administration" OR DE "DISTANCE education students" OR DE "DISTANCE education teachers" OR DE "ONLINE education" OR DE "OPEN learning" OR DE "TELECOMMUNICATION in education" OR DE "TELECOMMUTING" OR DE "TELECOURSES" OR DE "VIRTUAL schools") NOT TI universit* OR “higher education” OR postgrad* OR undergrad* OR “tertiary education” OR college OR campus* OR dent* OR nurs* OR pharmac* OR medic* OR "health professional*" OR "health worker*" OR "healthcare worker*" OR "health personnel" OR nonpharmaceutical OR energy OR pharmaceutical OR pharmacy OR clinic* OR pathology OR telemedicine OR inflammation OR patient* OR neurolog* OR telehealth OR surgery OR vaccin* OR antib* OR Disorder* OR Disabilit* OR Physical activit* OR Nutrition OR Diet* AND XApply equivalent subjects Limiters AND XDate Published: 20200101-20211231 AND Source Types XAcademic Journals AND Language Xenglish |

**Table B Summary of study appraisal from JBI appraisal checklist**

| Author | Study Design | Score based on appropriate JBI appraisal* | | | | | | | | | | Overall score (%) |
| --- | --- | --- | --- | --- | --- | --- | --- | --- | --- | --- | --- | --- |
|  |  | 1 | 2 | 3 | 4 | 5 | 6 | 7 | 8 | 9 | 10 |  |
| Beach KD et al. | Quasi-Experimental study | Y | Y | Y | N | Y | N | Y | U | Y | - | 66.7 |
| Çetin H & Türkan A | Quasi-Experimental study | Y | Y | Y | N | Y | N | Y | Y | Y | - | 77.8 |
| Christopoulos A & Sprangers P | Quasi-Experimental study | Y | U | Y | Y | Y | N | Y | N | Y | - | 66.7 |
| Cunha J et al. | Quasi-Experimental study | Y | U | Y | Y | N | Y | Y | N | Y | - | 66.7 |
| Fiş Erümit S | Qualitative study | Y | Y | Y | Y | Y | N | N | Y | N | Y | 70.0 |
| Gim N | Quasi-Experimental study | Y | Y | Y | N | N | Y | Y | Y | N | - | 66.7 |
| Huertas-Abril CA | Quasi-Experimental study | Y | Y | Y | N | N | N | Y | Y | Y | - | 66.7 |
| Ilhan GO, Kaba G & Sin M | Quasi-Experimental study | Y | Y | Y | N | Y | Y | Y | Y | Y | - | 88.9 |
| Kiili K et al. | Quasi-Experimental study | Y | Y | Y | N | Y | U | Y | Y | Y | - | 77.8 |
| Loukomies A & Juuti K | Qualitative study | Y | Y | Y | Y | Y | N | Y | Y | Y | Y | 90.0 |
| Meeter M | Quasi-Experimental study | Y | Y | Y | N | Y | N | Y | Y | Y | Y | 80.0 |
| Panskyi T et al. | Quasi-Experimental study | Y | U | N | Y | N | U | Y | Y | Y | - | 55.6 |
| Park S & Kim S | Quasi-Experimental study | Y | Y | Y | N | N | Y | Y | Y | Y | - | 77.8 |
| Simpson JC | Cross-sectional study | Y | Y | N | Y | N | N | N | N | - | - | 37.5 |
| Spitzer MWH & Musslick S | Quasi-Experimental study | Y | Y | Y | Y | N | U | Y | N | Y | - | 66.7 |
| Stalin LT & Kim Hua T | Quasi-Experimental study | Y | Y | Y | N | Y | Y | Y | N | Y | - | 77.8 |
| Tajik F & Vahedi M | Cross-sectional study | N | N | N | N | N | N | Y | Y | - | - | 25.0 |
| Wang D et al. | Cross-sectional study | Y | N | N | N | N | N | N | N | - | - | 12.5 |
| Wang X et al. | Cross-sectional study | Y | U | Y | Y | N | N | Y | Y | Y | - | 66.7 |
| Wijaya TT | Cross-sectional study | N | N | N | N | N | N | Y | N | - | - | 12.5 |
| Xie ZY et al. | Cross-sectional study | N | Y | Y | N | Y | Y | Y | Y | - | - | 75.0 |
| Yen ELY & Mohamad M | Quasi-Experimental study | Y | Y | Y | Y | Y | Y | Y | Y | N |  | 88.9 |

Note: Y=Yes; N=No; U=Unclear
